# Salt tolerance at single cell level in giant-celled Characeae

**DOI:** 10.3389/fpls.2015.00226

**Published:** 2015-04-28

**Authors:** Mary J. Beilby

**Affiliations:** Plant Membrane Biophysics, Physics/Biophysics, School of Physics, University of New South Wales, Sydney, NSWAustralia

**Keywords:** Characeae, salt tolerance, electrophysiology, current-voltage characteristics, action potentials, proton pump, H^+^/OH^-^ channels, non-selective cation channels

## Abstract

Characean plants provide an excellent experimental system for electrophysiology and physiology due to: (i) very large cell size, (ii) position on phylogenetic tree near the origin of land plants and (iii) continuous spectrum from very salt sensitive to very salt tolerant species. A range of experimental techniques is described, some unique to characean plants. Application of these methods provided electrical characteristics of membrane transporters, which dominate the membrane conductance under different outside conditions. With this considerable background knowledge the electrophysiology of salt sensitive and salt tolerant genera can be compared under salt and/or osmotic stress. Both salt tolerant and salt sensitive Characeae show a rise in membrane conductance and simultaneous increase in Na^+^ influx upon exposure to saline medium. Salt tolerant *Chara longifolia* and *Lamprothamnium* sp. exhibit proton pump stimulation upon both turgor decrease and salinity increase, allowing the membrane PD to remain negative. The turgor is regulated through the inward K^+^ rectifier and 2H^+^/Cl^-^ symporter. *Lamprothamnium* plants can survive in hypersaline media up to twice seawater strength and withstand large sudden changes in salinity. Salt sensitive *C. australis* succumbs to 50–100 mM NaCl in few days. Cells exhibit no pump stimulation upon turgor decrease and at best transient pump stimulation upon salinity increase. Turgor is not regulated. The membrane PD exhibits characteristic noise upon exposure to salinity. Depolarization of membrane PD to excitation threshold sets off trains of action potentials, leading to further loses of K^+^ and Cl^-^. In final stages of salt damage the H^+^/OH^-^ channels are thought to become the dominant transporter, dissipating the proton gradient and bringing the cell PD close to 0. The differences in transporter electrophysiology and their synergy under osmotic and/or saline stress in salt sensitive and salt tolerant characean cells are discussed in detail.

## Introduction

### Advantages of Characeae Experimental System for Salinity Studies

#### Large Cell Size and Simple Morphology

**FIGURE 1:**
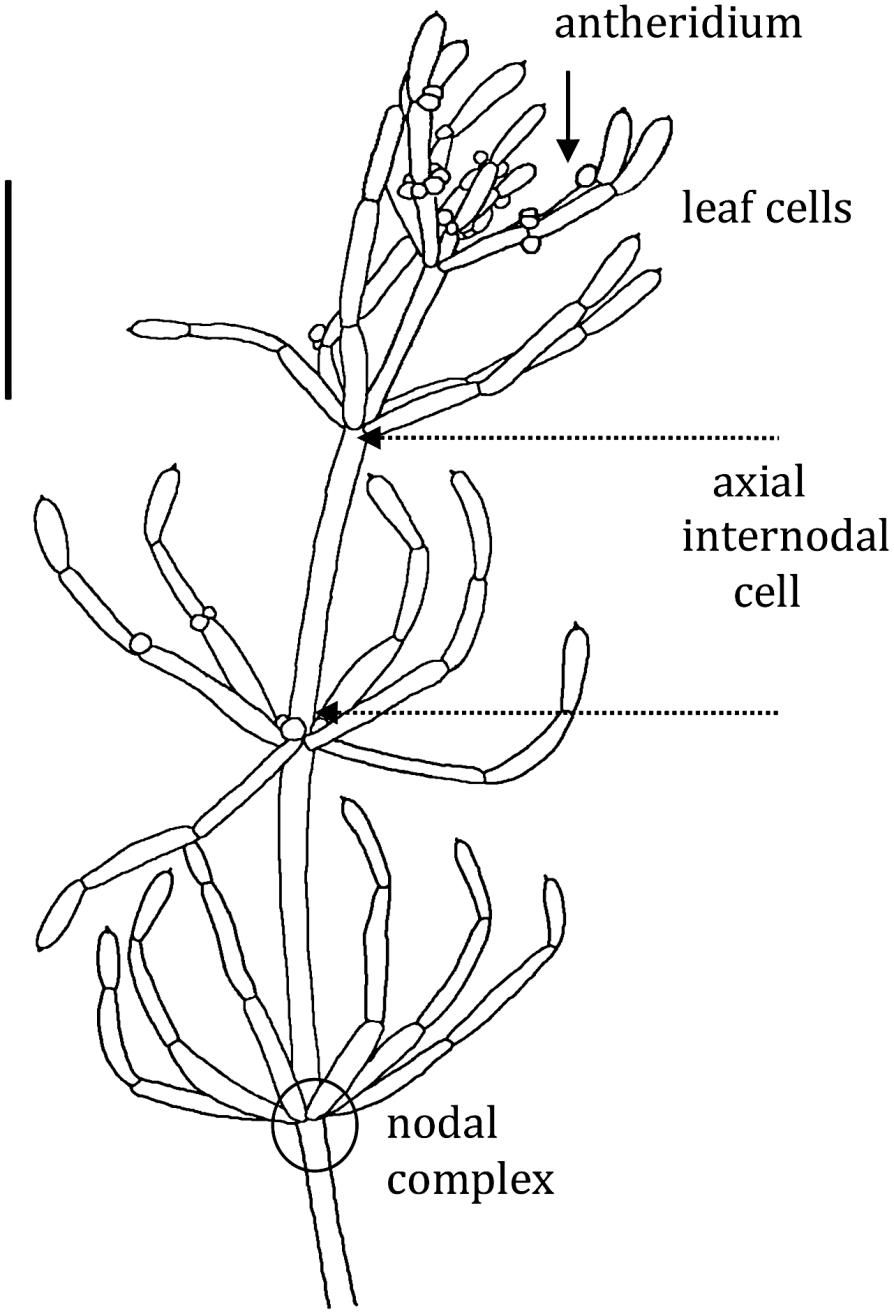
***Chara australis* male plant: each segment is a single cell**. Both leaf cells and axial internodes can be excised from the plant for experiments. The excised cells survive and new plants regenerate from small cells (not shown) in each nodal complex. Only the axial internodes near the top of the plant grow. The male reproductive structures, antheridia, can be found at the base of the whorl and branch-let nodes. The bar represents 10 mm (drawn by Michelle Casanova – adapted from Figure 1.6 of Beilby and Casanova, 2013).

The thallus of characeaen plant consists of stems (axes), which are made of long multinucleate single cells interrupted by multicellular nodes. The nodes also give rise to branch-lets, which are similar to leaves of higher plants, but also consist of single cells (see **Figure 1**). The axial internode cell can be up to 1 mm in diameter and several cm long. The plants have colorless rhizoids instead of roots and these are also large cells joined end to end. The axial or leaf cells survive excision from the plant and can regenerate new plants from the adjacent nodal complexes. These excised cells can be used in prolonged experiments (up to 24 h). Pioneering electrical and transport measurements were performed on the characean plants (Walker, 1955; Hope and Walker, 1975; Beilby and Casanova, 2013).

#### Position on Phylogenetic Tree

Recent phylogenetic studies (Karol et al., 2001) have shown that charophytes (that contain the Characeae family) are the closest living relatives of the ancestors of all land plants. Land plants emerged onto land ∼470 million years ago (Domozych et al., 2012), altering the atmosphere, reshaping the geology and enabling the evolution of terrestrial animals (Sorensen et al., 2010). While Characeae are now thought to be less closely related to land plants than another charophyte group Zygnematales (Wodniok et al., 2011; Timme et al., 2012), they are still positioned at the origin of land plants. Consequently, the large body of electrophysiological and physiological data provides valuable insights into many aspects of higher plants and into plant evolution (Beilby and Casanova, 2013). The question whether common ancestors of Characeae and land plants lived in freshwater or marine environments remains open (Graham and Gray, 2001; Kelman et al., 2004) as characean fossils were found in sediments from brackish and marine habitats (Martin et al., 2003). The transition of plants to land would have been less challenging from freshwater, as marine algae would have faced desiccation in air as well as hypersalinity in drying saline pools (Raven and Edwards, 2001). Further, fresh water plants would have already developed roots/rhizoids to acquire nutrients from the soil in the oligotrophic environment (Rodriguez-Navarro and Rubio, 2006).

#### Salt Tolerant and Salt Sensitive Genera

The salt tolerance or sensitivity of the extant Characeae mirrors that of land plant glycophyte–halophyte distribution: majority live in fresh water and only few species are truly salt tolerant. The salt tolerant Characeae include some *Tolypella*, some *Chara*, and all *Lamprothamnium* species. The most salt tolerant species respond to salinity changes by complete turgor regulation through changing vacuolar concentrations of K^+^, Cl^-^ and sometimes Na^+^ or sucrose: *Tolypella nidifica* and *glomerata* (Winter et al., 1996), *Chara longifolia* (Hoffmann and Bisson, 1986), and all *Lamprothamnium* species (Bisson and Kirst, 1980a; Okazaki et al., 1984; Beilby et al., 1999; Casanova, 2013; Torn et al., 2014). The salt tolerance of *Lamprothamnium* is remarkable: plants with reproductive organs were found in Australian lakes at up to twice the salinity of seawater (Burne et al., 1980; Williams, 1998). *C. australis* or *corallina* and *Nitella flexilis*, on the other hand, are obligate freshwater species that regulate their internal osmotic pressure (Gutknecht et al., 1978; Sanders, 1981; Bisson and Bartholomew, 1984). *C. australis* plants exhibit 100% mortality after ∼5 days in media containing 100 mM NaCl and 0.1 mM Ca^2+^ (Shepherd et al., 2008).

##### Components of saline stress

To resolve different components of salinity stress, cells can be exposed to a step up in osmolarity by employing sorbitol medium (for instance), followed by isotonic saline solution. Such experiments facilitate the measurement of short term defensive and stress responses to each component in dose dependent manner. The interpretation of results must allow for long term effects that might be due to slow acting mechanisms, such as compatible solute production and gene expression. For instance, Kanesaki et al. (2002) found that salt stress and hyperosmotic stress resulted in different gene expression in the ancient cyanobacteria *Synechocystis*.

The osmotic stress and Na^+^ toxicity require different types of sensors and defensive mechanisms. The increase in osmolarity of the outside medium decreases the water potential and water flows out of the cell within seconds of exposure (Steudle and Zimmermann, 1974). The turgor of the cell drops, limiting growth, making cells prone to injury and affecting photosynthetic activity (Allakhverdiev et al., 2000; Allakhverdiev and Murata, 2008). To control turgor, cells have to be able to sense it. The turgor sensors are still being identified (Boudsocq and Lauriere, 2005).

The increase in NaCl concentration presents another problem. Characean cells are not very permeable to Cl^-^, but Na^+^ rapidly floods the cells through non-selective cation channels (NSCCs; Demidchik and Maathuis, 2007). Cell has to expend energy to move Na^+^ from the cytoplasm, where it replaces K^+^ and inhibits metabolic functions. In cyanobacteria *Synechococcus* increased Na^+^ medium concentration caused slow and irreversible inactivation of the photosystems (Allakhverdiev et al., 2000).

The Characeae plants are totally submerged in the medium and cannot fight salinity by forming salt glands or exporting salt to sacrificial tissues, or blocking salt movement into the shoot: every single cell in the plant has to be salt tolerant. Thus, comparing the electrophysiology of salt tolerant and salt sensitive characean species is likely to identify a minimal ensemble of factors that bestow salt tolerance at cellular level. This review reports on the progress of such studies.

## Experimental Techniques

Characeae experimental system is unique by providing the comparison between the intact cell and the preparations with escalating interventions. The methodology adapted to these large celled plants is briefly summarized below.

The cytoplasmic layer of a single cell is up to 10 μm thick and the vacuole occupies 95% of the cell volume (Raven, 1987; Beilby and Shepherd, 1989). Microelectrodes can be positioned in the cytoplasm as well as in the vacuole, and membrane potential difference (PD) is measured separately across plasma membrane and tonoplast (Findlay and Hope, 1964; Beilby, 1989). However, the cytoplasm often excludes microelectrodes (Walker, 1955) and the cells become more prone to damage upon electrode re-insertion. The access to the cytoplasmic compartment can be improved by gentle centrifugation that moves cytoplasm to one end of the internode (Hirono and Mitsui, 1981; Beilby and Shepherd, 1989). The electrode is impaled before the streaming cytoplasm redistributes along the cell, or the cell can be wilted in the air and the cytoplasmic plug tied off, preparing cytoplasm-enriched fragments (Hirono and Mitsui, 1981; Beilby and Shepherd, 1989). Alternatively, cell ends can be cut and perfusion medium replaces the vacuolar sap (Tazawa, 1964). Rapid perfusion rate or inclusion of EGTA in the perfusion medium disintegrates the tonoplast (Williamson, 1975; Tazawa et al., 1976). This preparation not only makes the plasma membrane accessible, but also surrounds it with media of known composition. To study the tonoplast the plasma membrane is permeabilized using EGTA (Shimmen and Tazawa, 1982; Tester et al., 1987). The tonoplast also surrounds cytoplasmic droplets formed by cutting an internodal cell and immersing the end in vacuolar sap like medium (Luhring, 1986). While some of the latter techniques allow experimenters greater control over the surroundings of each membrane, they also perturb the living cell and may introduce artifacts.

The size of characean cells facilitated early water permeability measurements (Wayne and Tazawa, 1990), using transcellular osmosis technique. The internodal cell was placed in two-compartment chamber with media of different osmolarity in each chamber. The reversible partial block by mercury derivatives suggested that some of the water moves across the membrane through water channels aquaporins. Pressure probe single cell measurements confirmed presence of aquaporins, although fraction of water and uncharged solutes permeating through them (as opposed to lipid bilayer) is still under consideration (Henzler and Steudle, 1995; Schutz and Tyerman, 1997). Ye et al. (2004, 2005) formulated tension/cohesion model for closure of water channels with increasing osmolarity. Henzler et al. (2004) found that water channels also close in response to oxidative stress.

The size of the cells can be a disadvantage when the membrane PD is controlled by voltage clamp. A longitudinal wire electrode or a small central compartment in a multi-compartment cell holder may be employed to space clamp the cell: to pass uniform currents and avoid conductance “hot spots” (Smith, 1984a; Beilby, 1990). To obtain current-voltage (I/V) characteristics of the plasma membrane (or both membranes in series), the membrane PD is clamped to bipolar staircase (Gradmann et al., 1978). The alternating short excursions to PD levels above and below the resting PD avoid prolonged large currents and transport number effects (accumulation of ions near the membrane outer surface – see Beilby, 1990). I/V profiles over wide PD windows (up to 500 mV) can be obtained in less than 10 s (Beilby, 1989). However, it is necessary to check the raw data to insure that the current has leveled at the end of each pulse and that it returned to near zero while the membrane PD was clamped at the resting level. Long time current dependencies need to be investigated separately by prolonged (seconds) voltage clamp to different PD levels.

## Background of Characeae Electrophysiology

Thousands of I/V profiles have now been recorded on various Characeae species and under a range of conditions. The results are mostly consistent, suggesting that the plasma membrane can take on different states, depending on the outside conditions. This large body of data allows identification of ion transporter I/V profiles, their responses and synergies at the time of abiotic stress (such as salinity increase), as well as comparison to transporters of higher plants. The forthcoming sequencing of Characeae (Stefan Rensing, personal communication) will allow even more detailed comparisons on molecular level.

**FIGURE 2:**
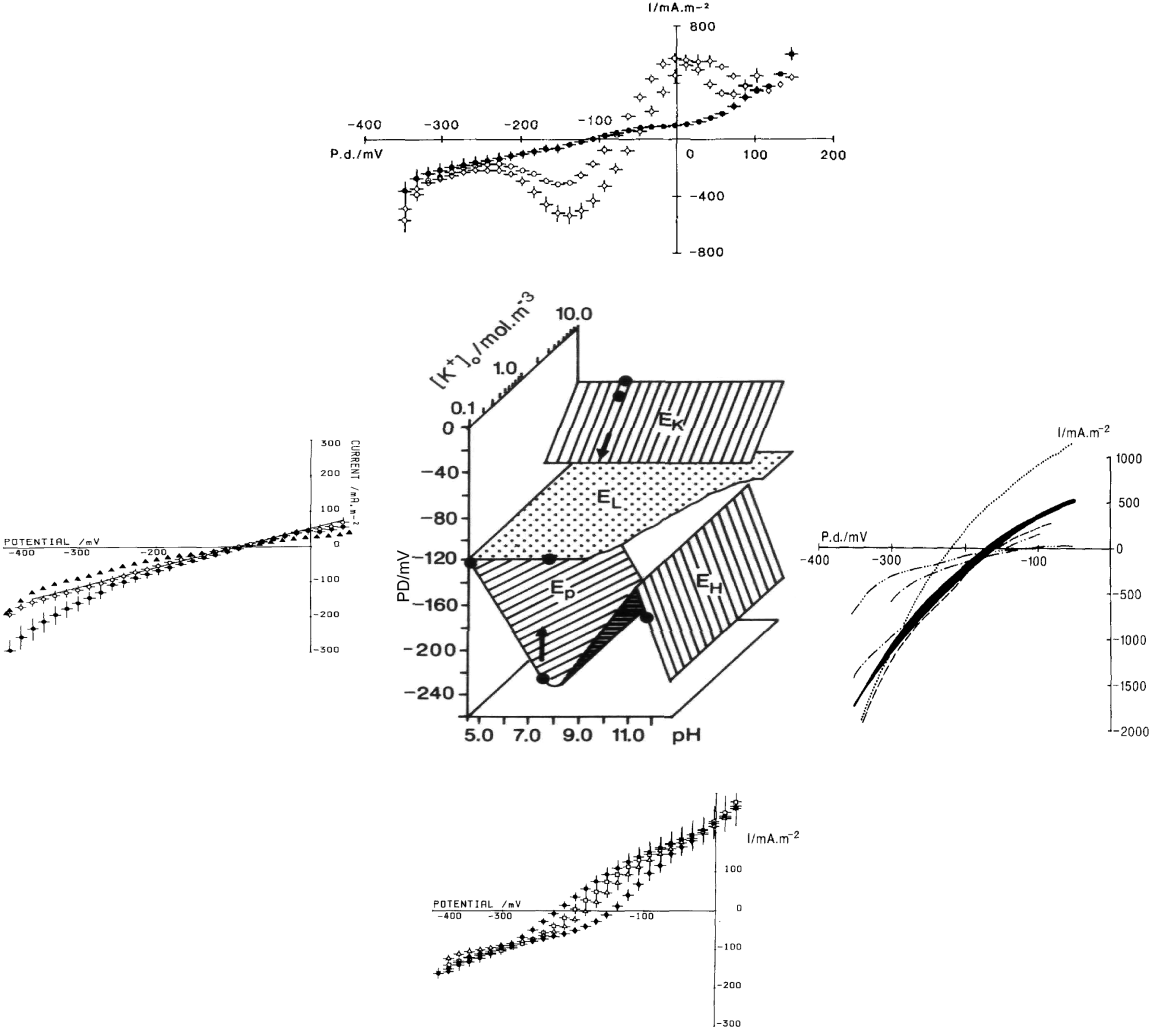
**Different states of plasma membrane: central panel shows the membrane PD in different states as function of pH_o_ and [K^+^]_o_ (E_p_ reversal PD of the proton pump, E_L_ reversal PD for the background/leak current, E_K_ Nernst PD for K^+^, E_H_ Nernst PD for H^+^ or OH^-^)**. The figure is based on **Figure 1** (Beilby, 1990) with the I/V characteristics updated from later publications. Bottom panel: proton pump-dominated state at pH_o_ of 4.5 ♦, 5.5 Δ, 6.5 □, and 7.5 • (Beilby, 1984). E_p_ is most negative at pH_o_ 7–8 (shown as a dot on the “pump surface”) and tends toward E_L_ at pH 4.5 (a point where the pump and background surfaces meet). Left panel: background state with 10 I/V runs summarized from nine cells exposed to DES (diethyl stilbestrol). These cells stabilized after 30 min DES exposure (shown by a point on the background surface). Four La^3+^-treated cells exposed to DES for 30 min, •, continued to change as shown by a single I/V run on La^3+^-treated cell, with DES exposure of I hr 15 min (Beilby, 1984). Top panel (Beilby, 1986): I/V characteristics of cells in K^+^ state, summary from seven cells in 5 mM K^+^ APW 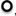 10 mM K^+^ APW 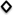 (the two points on K^+^ surface) and 0.1 mM K^+^ APW • (here the K^+^ channels closed revealing the background state, see the arrow in central part of figure). Right panel (Beilby and Bisson, 1992): high pH state: pH 11.5 (dotted line), pH 10.5 – two I/V runs in fast succession shown by black shading, pH 10.5 + 2.5 mM Na_2_SO_4_ (dashed line), back to pH 10.5 (dash, two dots, dash line), pH 10.5 + 10 mM Na_2_SO_4_ (dash dot dash line), and finally back to pH 10.5 (dash, three dots, dash line). The I/V characteristics in this state can be quite variable. As NaOH (5–30 mM) was used to bring the APW to high pH, the effect of Na^+^ concentration increase was explored. Beilby and Bisson (1992) did not find a consistent effect, but high concentrations might affect the H^+^/OH^-^ channel activation via ROS response, see text.

There are many ion transporters in both plasma membrane and the tonoplast and new ones are being discovered. However, only a small number of transporter types dominate the membrane conductance and the I/V profiles. The I/V characteristics can change substantially depending on the pH and K^+^ concentration of the outside medium (see the central part of **Figure 2**).

### Pump State

**FIGURE 3:**
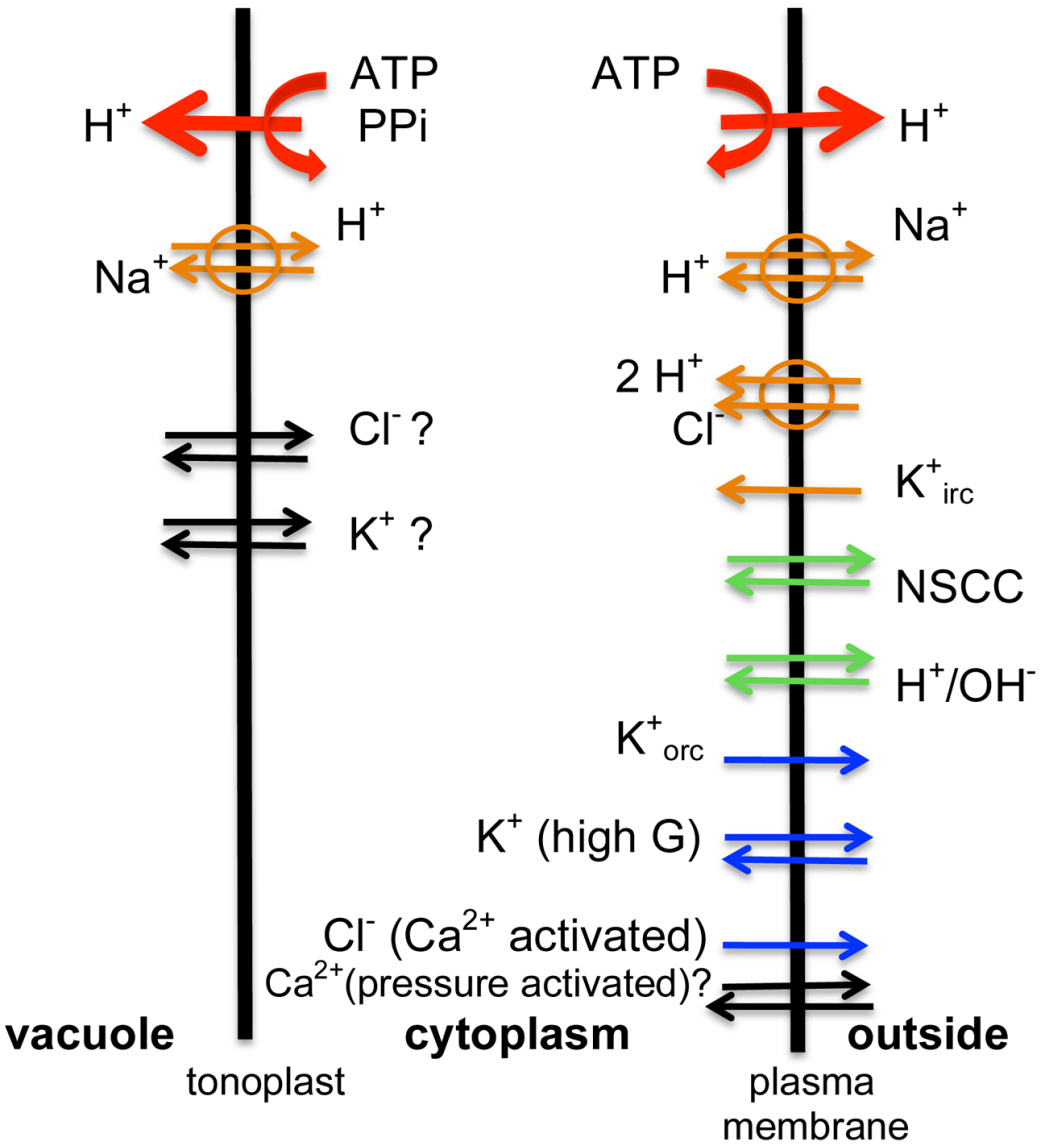
**The transporter scheme for the characean cell: same types of transporters are found in both salt tolerant and salt sensitive genera**. The energizing ATPases and PPases (red arrows) drive H^+^ out of the cytoplasm. The proton motive force is employed by Na^+^/H^+^ aniporters and 2H^+^/Cl^-^ symporter, while negative PD opens K^+^ inward rectifier (orange arrows). In salt sensitive Characeae the pumps are not stimulated by low turgor and fail in saline media. In normal pond water NSCC channels bring in nutrients and H^+^/OH^-^ channels aid photosynthesis by exporting OH^-^ in alkaline bands (green arrows). In time of saline stress Na^+^ enters through NSCC channels in all Characeae, but salt tolerant Characeae keep the Na^+^/H^+^ antiporters going and prevent global opening of H^+^/OH^-^ channels. The outward rectifier, the high conductance K^+^ channels or Ca^2+^ activated Cl^-^ channels are not active in steady state (blue arrows). The inflow of Ca^2+^ into the cytoplasm (black arrows) at the time of AP or hypoosmotic regulation is well documented, but the sources (outside, internal stores in the cytoplasm or the vacuole) are still disputed and beyond the scope of this article. Similarly, there are K^+^ and Cl^-^ transporters on the tonoplast (black arrows), but discussion of these is beyond the scope of this article. The Ca^2+^-activated Cl^-^ channels provide the depolarizing phase of the AP with the outward rectifier contributing to the recovery of resting PD. In salt tolerant Characeae the Ca^2+^-activated Cl^-^ channels and high conductance K^+^ channels mediate hypoosmotic regulation.

At neutral to slightly alkaline pH_o_ with K^+^ below 1 mM (a typical fresh water pond, where majority of characean species live), the plasma membrane resting PD is quite negative at -200 to -250 mV. The I/V characteristics exhibit a beautiful sigmoid shape generated by the energizing export of protons from the cytoplasm by the proton ATPase (see the bottom part of **Figure 2** and **Figure 3**). For history of the characean proton pump research and modeling with the cyclic enzyme-mediated HGSS model (Hansen et al., 1981) see chapter 2 of Beilby and Casanova (2013).

### Background State

If the pump is turned off by some metabolic inhibitors or by circadian rhythms, the underlying null or background state is revealed: more depolarized resting PD near -100 mV and linear I/V profile in the PD window of ∼ +50 to ∼-350 mV (see the left part of **Figure 2**). The background current was fitted by an empirical equation:

$$I_{background}=G_{background}\left({V-E}_{background}\right)$$

where G_background_ is PD independent conductance and E_background_ (E_L_ in **Figure 2**) is the reversal PD chosen as -100 (± 20) mV (-120 mV in **Figure 2**). This value was derived experimentally and its origin is still a puzzle. The background current is thought to flow through non-selective PD-independent cation channels (NSCC – Demidchik and Maathuis, 2007), which supply micronutrients to the plant and contribute to signaling (**Figure 3**).

At PD levels more negative than ∼-300 mV the background current is obscured by the inward rectifier current, K^+^_irc_, while at PD levels more positive than ∼ -50 mV the outward rectifier current, K^+^_orc_, predominates. The inward and outward rectifier currents, mainly carried by K^+^, were modeled by the Goldman-Hodgkin-Katz (GHK) equation, multiplied by the Boltzmann distribution of open probabilities to make the PD-dependence stronger (Beilby and Walker, 1996; Amtmann and Sanders, 1999). The early activation of inward rectifier K^+^_irc_ can be observed near -400 mV on the pump state curves (bottom of **Figure 2**) and K^+^ state curves (top of **Figure 2**). The early activation of outward rectifier, K^+^_orc_, can be found near 0 PD in pump state curves and at +50 mV in the K^+^ State curves.

The pump state and the underlaying background state are the native states for the salt sensitive Characeae in their low salt, slightly alkaline pond media.

### K^+^ State

As K^+^ concentration in the medium rises above ∼1 mM, large conductance K^+^ (high G K^+^) channels open, short-circuit the pump current and become the dominant membrane conductance (Oda, 1962; Smith and Walker, 1981; Sokolik and Yurin, 1981; Keifer and Lucas, 1982; Smith, 1984b; Beilby, 1985; Sokolik and Yurin, 1986; Tester, 1988a,b,c). The I/V characteristics of the K^+^ state are very distinct: the two regions of negative conductance arise from the strong PD dependence of the channels (see the top part of **Figure 2**). The I/V profiles can also be modeled by GHK equation supplemented with Boltzmann distribution of open probabilities (Beilby and Shepherd, 2001b). The K^+^ conductance increases with outside K^+^ concentration until it becomes so large that the cells cannot be voltage-clamped. This property of K^+^ state is exploited in K^+^ anesthesia technique of measuring membrane PD without inserted electrodes (Hayama et al., 1979). K^+^ channels are blocked totally and reversibly by tetraethylammonium (TEA) revealing the linear background state, which seems independent of K^+^ concentration (see the top part of **Figure 2**). The activation of high G K^+^ channels is observed in tissues, stomata and roots of land plants (Epstein, 1976; Cheeseman and Hanson, 1979; Blatt, 1988).

### High pH State

If pH of the medium rises above 9.0, the resting PD starts to follow the equilibrium PD for H^+^ or OH^-^ with resting PD more negative than -200 mV in some cells (Bisson and Walker, 1980). The membrane conductance can increase by up to 5 S.m^-2^. This H^+^/OH^-^ state is inhibited by darkness, photosynthesis inhibitor DCMU, various metabolic inhibitors (such as DES or DCCD) or lack of Ca^2+^ in the medium, which can be replaced by Mg^2+^ (Bisson, 1984). Beilby and Bisson (1992) found that the I/V characteristics exhibit slight downward curvature (see right part of **Figure 2**). However, the conductance and the reversal PD are quite variable. Al Khazaaly and Beilby (2012) modeled the I/V characteristics at high pH, using the GHK equation supplemented by the Boltzmann probability distribution. H^+^ or OH^-^ was tested as the transported ion: OH^-^ seems more probable, as the channel number multiplied by the channel conductance remains relatively constant throughout the pH range, whereas for H^+^ the same parameter had to be increased by several orders of magnitude to match the high conductance at very high pH (Beilby and Casanova, 2013). The high pH state is reversibly blocked by the zinc ion, potent inhibitor of animal H^+^ channels (Al Khazaaly and Beilby, 2012). The H^+^/OH^-^ channels participate in the pH banding pattern, where ring shaped zones of pump-dominated or H^+^/OH^-^ channel dominated membrane are set up by the cells to acquire DIC (dissolved inorganic carbon) and make photosynthesis more efficient (Hope and Walker, 1975; Beilby and Bisson, 2012; Beilby and Casanova, 2013).

In their normal habitats, the whole Characeae cells and plants are not exposed to pH of 10 and higher. However, due to export of OH^-^ in the alkaline bands, the external pH rises to 10 and above. The exposure of cells to high pH highlighted mechanisms leading to banding formation (Beilby and Bisson, 2012) and facilitated detailed description and modeling of the I/V characteristics of the H^+^/OH^-^ channels (Beilby and Bisson, 1992; Beilby and Al Khazaaly, 2009; Al Khazaaly and Beilby, 2012).

A detailed study of the I/V characteristics of *Lamprothamnium* cells acclimated to salinities ranging from 0.2 to full artificial sea water (ASW) showed that in the higher salinities (from ∼1/3 ASW) the cells could be found in three different resting states: pump state, background state or K^+^ state (Beilby and Shepherd, 2001a). Similarly to salt sensitive *C. australis* (left side of **Figure 2**), *Lamprothamnium* cells in the background state exhibited near linear I/V characteristics between -50 and -200 mV with reversal PD close to -100 mV.

## Surviving in Saline Media: Salt Tolerant Characeae

### Hyperosmotic Adjustment Mechanisms

Upon increase of osmolarity in the external medium, the water potential drops, water flows out of the cell and the turgor pressure decreases. To restore turgor the osmolarity of the vacuole must be increased. This process requires energy. The energizing elements of the ion transport are proton ATPases and PPases on the plasma membrane and the tonoplast (**Figure 3**). The main osmotica, K^+^ and Cl^-^ are imported through inward rectifier K^+^_irc_ and 2H^+^/Cl^-^ symporter (**Figure 3**). Such turgor adjustment is well documented in higher plants (for instance in *Arabidopsis* roots – Shabala and Lew, 2002). The Na^+^ inflow occurs through the NSCCs (Tester and Davenport, 2003; Demidchik and Maathuis, 2007). These channels can be partially blocked by high Ca^2+^ in the external medium (Tester and Davenport, 2003). The plant cells strive to keep low Na^+^ in the cytoplasm employing Na^+^/H^+^ antiporters at both membranes (Tester and Davenport, 2003 and **Figure 3**). Several of these mechanisms were initially discovered in the Characeae experiments.

### H^+^ Pump Activation

Bisson and Kirst (1980a,b) and Okazaki et al. (1984) observed hyperpolarization of membrane PD in *Lamprothamnium* sp. upon increase in salinity while the vacuolar concentrations of K^+^ and Cl^-^ increased to regulate turgor to ∼300 mosmol/kg (Bisson and Kirst, 1980b). Reid et al. (1984) resolved the response further into a transient PD depolarization for ∼10 min, followed by hyperpolarization. They also found a transient drop in ATP concentration paralleled by a rise in respiration. The cytoplasmic streaming speed diminished briefly and then increased, while the influxes of Na^+^, K^+^, and Cl^-^ increased over 800 min. Working on the only salt tolerant *Chara* species, *C. longifolia*, Yao et al. (1992) and Yao and Bisson (1993) demonstrated that the proton pumping also increased in more saline media and turgor was regulated: cell PD transiently hyperpolarized and conductance increased (95–144 h). Okazaki et al. (1984) challenged *Lamprothamnium* cells with a sorbitol hyperosmotic step and also observed membrane hyperpolarization. They suggested that it is the decrease in turgor, which initiates the turgor regulation observed by Bisson and Kirst (1980a). To confirm this hypothesis Al Khazaaly and Beilby (2007) compared the step from 1/6 ASW to 1/3 ASW in salinity and equivalent step in osmolarity using sorbitol (Sorbitol ASW). The I/V characteristics were measured for up to 7 h following either type of hyperosmotic step and the data were modeled to resolve the responses of various transporters. In both treatments the average cell PD hyperpolarized to similar level (∼-150 mV) through the activation of the proton pump and the inward rectifier channels were opened at more positive PDs. The authors also found that the cells in K^+^ state were able to switch to pump state upon turgor decrease or salinity increase, and were able to regulate turgor.

### Na^+^ Transport

**FIGURE 4:**
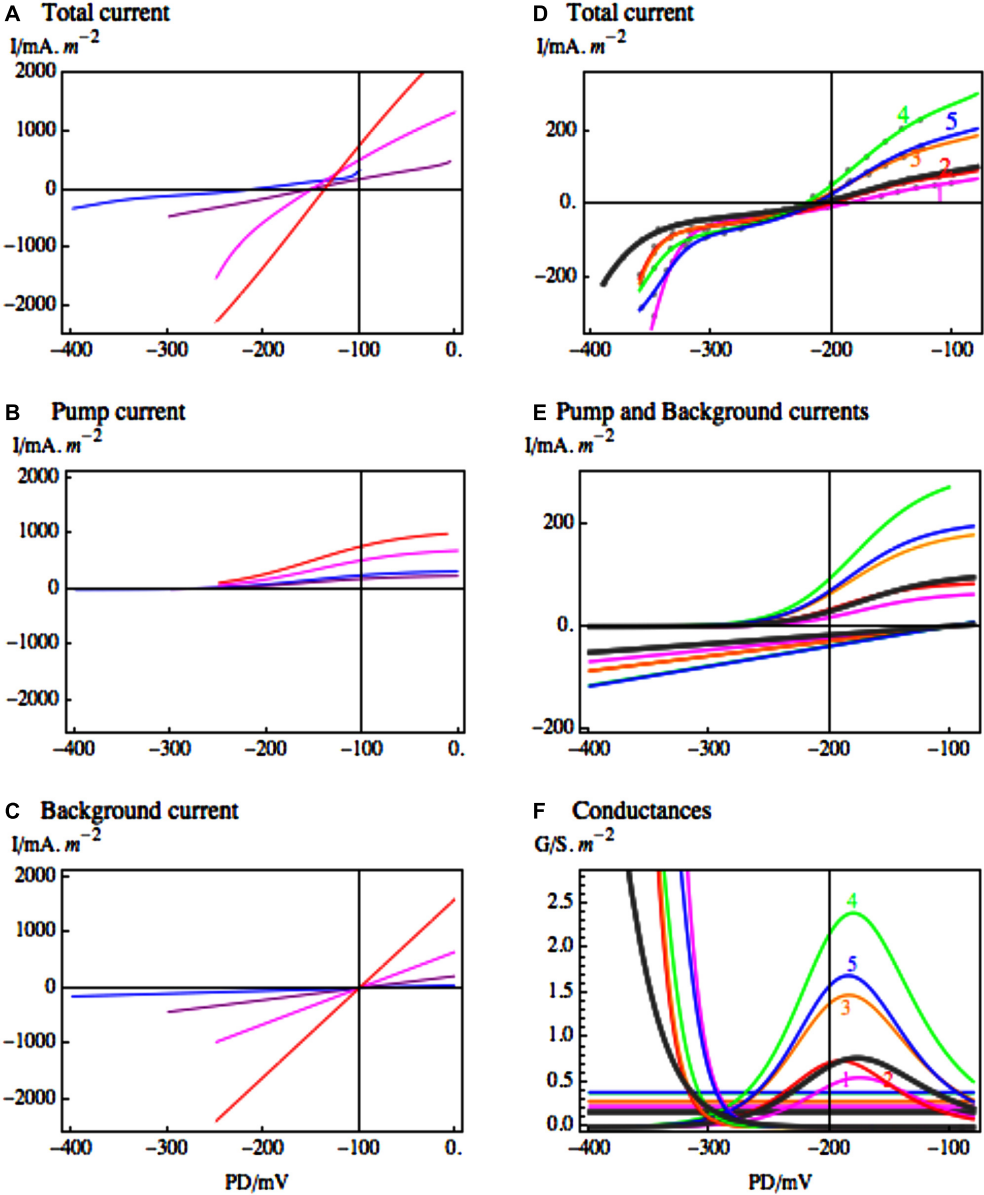
**The response of the most conductive transporters in *Lamprothamnium* to medium salinity. (A)** I/V characteristics of *Lamprothamnium* sp. in steady state, grown in media of increasing salinity: 0.2 seawater (SW), blue; 0.4 SW, purple; 0.5 SW, magenta; full SW, red. The currents have been fitted to data from 6 to 8 cells from each medium (adapted from Figure 6 of Beilby and Shepherd, 2001a). The fitted pump **(B)** and the background **(C)** current components are shown in same colors (for the fit parameters see Table 2 of Beilby and Shepherd, 2001a). The fitting of background current in media 0.4 – full SW was supported by comparison with cells in background state in each medium. **(D)** Characteristics from one *Lamprothamnium* cell acclimated to 0.2 SW and challenged by doubling salinity to 0.4 SW. Steady state I/V in 0.2 SW: black thick line; curve 1, magenta, 5 min of 0.4 SW; curve 2, red, 21 min of 0.4 SW; curve 3, orange, 41 min of 0.4 SW; curve 4, green, 2 h 34 min of 0.4 SW; curve 5, blue, 3 h 30 min of 0.4 SW (adapted from Figure 7 of Beilby and Shepherd, 2001a). The fitted pump and background current components **(E)** are shown in same colors. The conductances of the fitted current components, including the inward rectifier, are displayed in **(F)** also in same colors (for fit parameters see Table 3 of Beilby and Shepherd, 2001a).

Kishimoto and Tazawa (1965), early pioneers of the I/V technique, measured increased *Lamprothamnium* cell membrane conductance with rising salinity of the medium. However, modeling of the currents through the different transporters was necessary to resolve the increase of pump conductance and the conductance due to Na^+^ inflow. The *Lamprothamnium* cells in the background state showed clearly that the slope (conductance) of the I_background_ increased with medium salinity, while E_background_ remained close to -100 mV (Beilby and Shepherd, 2001a and **Figure 4C**). These cells confirmed the correct modeling of this underlying state for cells in pump state and K^+^ state (Beilby and Shepherd, 2001a). Al Khazaaly and Beilby (2007) confirmed that after exposure to Sorbitol ASW the background conductance actually decreased slightly, while in 1/3 ASW the background conductance increased by about a third.

Working on *C. longifolia* the Bisson group found that the Na^+^/Ca^2+^ ratio is important for turgor regulation (Hoffmann and Bisson, 1988; Hoffmann et al., 1989). The freshwater-incubated cells could only survive salinity increase and regulate turgor if the ratio was 10:1. Interestingly, the cells acclimated to their native medium (∼100 mM Na^+^ and 100 mM Mg^2+^ with Cl^-^ and SO_4_^2-^ as main anions) did survive higher Na^+^/Ca^2+^ ratios. Higher Ca^2+^ in the medium partially blocks the NSCC channels and diminishes the Na^+^ inflow.

Tester and Davenport (2003) estimated that cytoplasm would equalize with the external medium of 50 mM NaCl in 3 min, if there was no Na^+^ eﬄux. To frustrate the electrophysiologist Na^+^/H^+^ antiporter is electrically silent and consequently independent of membrane PD. Thermodynamically, the Na^+^/H^+^ anti-porter is an example of a Maxwell’s demon. Protons are pumped out of the cytoplasm to create inward proton gradient and negative membrane PD, Na^+^ flows in passively and is then removed by a “swap” for a proton. The primary energizing process is the proton pumping with ATP consumption as the cost to the cell (**Figure 3**).

Whittington and Bisson (1994) employed isotope ^22^Na^+^ to measure Na^+^ influx and eﬄux in salt sensitive *C. corallina* and fresh water-grown salt tolerant *C. longifolia* under mild salt stress of 20 mM NaCl. They found lower influx in salt tolerant *C. longifolia.* The addition of 1 mM Ca^2+^ to the medium greatly reduced influx into *C. corallina*. Na^+^ eﬄux was greater in *C. longifolia* and increased in both Characeae as pH_o_ changed from 7 to 5. In both species the eﬄux was unchanged by pH increase from 7 to 9. The eﬄux was not significantly inhibited by the Na^+^/K^+^ pump inhibitor amiloride, confirming the absence of such pump. *C. longifolia* grown in native saline medium (130 mM Na^+^ and 110 mM MgSO_4_) exhibited greater Na^+^ eﬄux, especially at pH_o_ 5 (Kiegle and Bisson, 1996). At pH 9 the calculated Δμ_H_ was not sufficient to drive the eﬄux and the authors concluded that there might be other mechanisms for Na^+^ eﬄux.

Davenport et al. (1996) found that despite surviving in salinities at seawater level and above, *Lamprothamnium* is also dependent on Ca^2+^ concentration of the medium. At low Ca^2+^ Na^+^ influx increased and the cells died. For both *Lamprothamnium* and *Chara australis* influx of ∼300 nmolm^-3^s^-1^ seems to be the limiting level for survival. The authors showed convincingly that low turgor promotes greater Na^+^ influx. Reduction of turgor by addition of up to 100 mM mannitol doubled the influx even at low medium concentration of 3.5 mM NaCl. Conversely, increasing the turgor of *Chara* cells by soaking them in concentrated KCl decreased the Na^+^ influx.

### Modeling Transporter Response to Salinity

Resolving the different transporter populations by modeling the I_Total_/V characteristics provides quantitative estimates of response to salinity/osmolarity increase. In *Lamprothamnium* plants acclimated to range of salinities the conductance of the background state increased with salinity from 0.5 S.m^-2^ in 0.2 ASW to 22.0 S.m^-2^ in full ASW (see **Figure 4C**). The cells in pump state increased pump currents in more saline media (see **Figure 4B**) with conductance maxima at 2 S.m^-2^ in 0.2 ASW and 5 S.m^-2^ in full ASW. But even with greater proton pumping the cell resting PDs became more depolarized with rising salinity: below -200 mV in 0.2 ASW to ∼-140 mV in full ASW (see **Figure 4A**). **Figures 4A–C** contain important message: *Lamprothamnium* cell can sense Na^+^ concentration in the medium and adjust pump activity to counteract the increased background conductance. In higher salinity the cell has to expend more energy powering the proton pump. The cells in K^+^ or background state could be “resting,” saving energy temporarily, while the proton gradient runs down. Al Khazaaly and Beilby (2007) demonstrated that these cells are able to “switch” the pump back on. It might be interesting to find out if there is a periodic (perhaps circadian) pattern for the different states to dominate.

Beilby and Shepherd (2001a) also modeled transient changes of the *Lamprothamnium* proton pump after salinity step of 150 mOsmol.kg^-1^, starting in dilute medium of 0.2 ASW (see **Figures 4D–F**). There was an initial decrease in the proton pump current and peak conductance (see **Figures 4D,E**, curve 1 magenta), coinciding with the low ATP concentration found by Reid et al. (1984). Then both the current and peak conductance increased, coming to a maximum after ∼2 h of hyperosmotic challenge (see **Figures 4D,E**, curve 4, green). Inward rectifier current responded within minutes of salinity increase by activating at more positive PDs (**Figures 4D,F**).

Thus the more researched salt tolerant Characeae, *Lamprothamnium sp.* and *Chara longifolia*, both exhibit proton pump stimulation upon salinity increase to keep the membrane PD negative, despite partial short-circuit of greater background conductance of Na^+^ inflow through NSCC channels. This response is crucial, as the proton pump provides the energy source for both up-regulation of turgor and prevention of toxic built up of Na^+^ in the cytoplasm (**Figure 3**). The membrane PD more negative than E_K_ facilitates the import of K^+^ through the inward rectifier channels to maintain K^+^/Na^+^ ratio supportive to normal enzyme function. K^+^ is also transported into the vacuole for turgor regulation. The import of Cl^-^ into the vacuole upon salinity/osmolarity increase is mediated by the 2H^+^/Cl^-^ symporter at plasma membrane, again powered by the proton electrochemical gradient (Sanders, 1980; Beilby and Walker, 1981). The Cl^-^ transporters at the tonoplast also have to work “uphill” to achieve the observed vacuolar concentrations (Teakle and Tyerman, 2010).

### Hypoosmotic Adjustment Mechanisms

Plants living in saline media have to cope with salinity changes: osmolarity of a shallow pond can drop in minutes in a torrential downpour. Cells have been observed to explode if the turgor became too great. What are the mechanisms for such sudden downward turgor adjustment? Working with different starting media and different salinity/osmolarity decrease in *Lamprothamnium* or *C. longifolia*, several investigators found rapid membrane depolarization to ∼ -70 mV for 30–60 min, accompanied by conductance rise up to an order of magnitude, with subsequent partial repolarization (Reid et al., 1984; Okazaki et al., 1984; Hoffmann and Bisson, 1990; Okazaki and Iwasaki, 1992; Beilby and Shepherd, 1996).

Okazaki and Tazawa (1986a,b) also observed streaming inhibition for up to 20 min upon hypoosmotic challenge. Low Ca^2+^ medium or presence of Ca^2+^ antagonist nifedipine abolished the depolarization, conductance increase and turgor regulation. Okazaki and Tazawa (1987) visualized the Ca^2+^ increase in the cytoplasm using fluorescence techniques: in ASW with normal high Ca^2+^ content fluorescence increased after about 1 min of hypoosmotic stress. If the cell was given a hypoosmotic shock in low Ca^2+^ ASW and Ca^2+^ increased later, the fluorescence rose immediately. The authors concluded that turgor mediated opening of Ca^2+^ channels has a small delay. Okazaki et al. (2002) made more detailed measurements of Ca^2+^ concentration in *Lamprothamnium* under hypoosmotic stress. After initial rapid increase from resting value of 100 nM to peak of 600 nM, the concentration dropped at 0.9 nM/s. They also estimated that maximum conductance was reached by 300 nM, but 400–600 nM was necessary for cytoplasmic streaming cessation.

**FIGURE 5:**
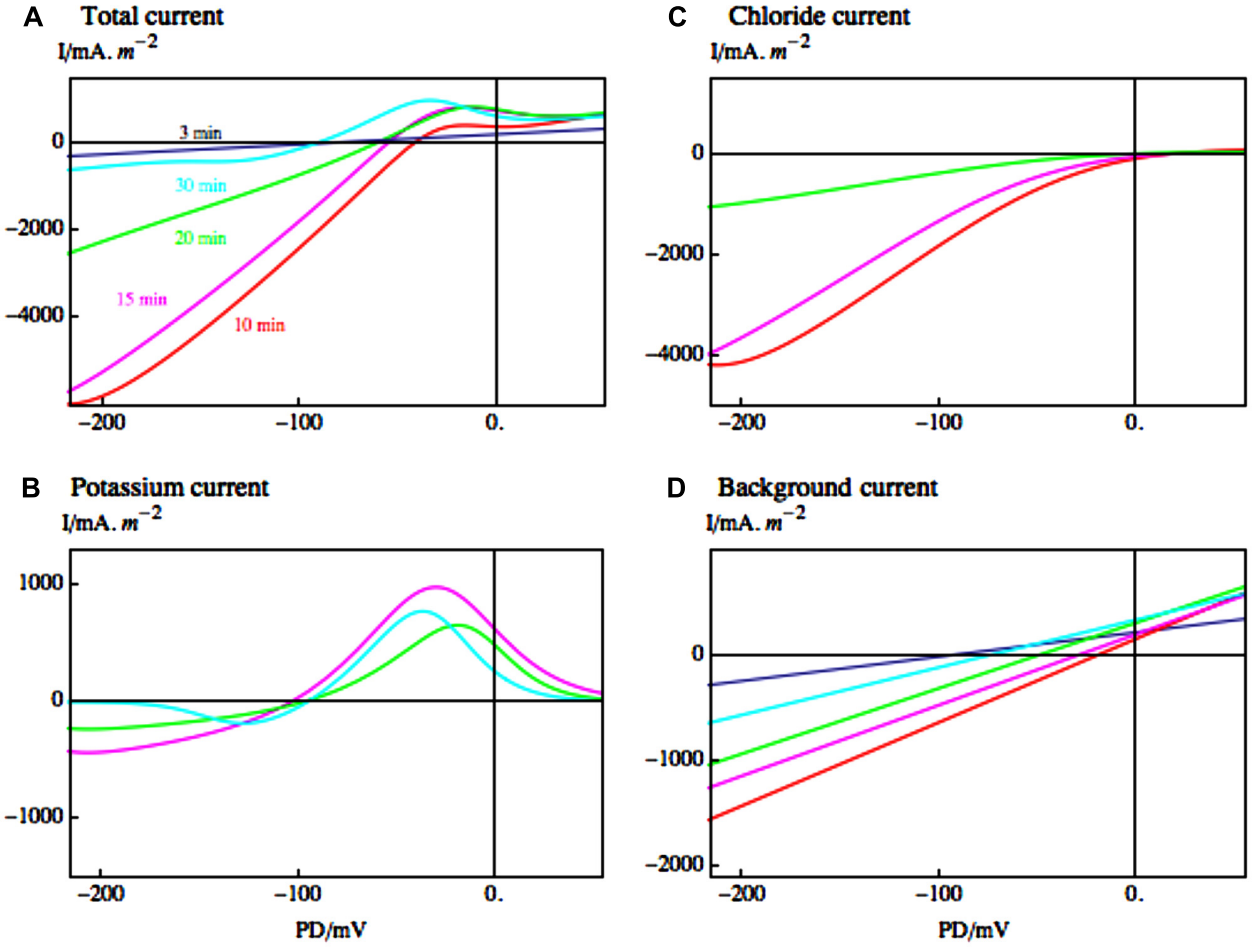
**Electrophysiology of the hypoosmotic regulation upon dilution step from 1/3 ASW to 1/6 ASW. (A)** The I/V characteristics are compiled from currents fitted to cells with Cl^-^ current blocked by exposure to LaCl_3_ or K^+^ current blocked by TEA at times: 3 min (dark blue); 10 min (red); 15 min (magenta); 20 min (green); 30 min (blue; Beilby and Shepherd, 1996, 2001b). **(B)** The fitted Cl^-^ currents are shown with the same types of line as in **(A)**. These currents appear with a slight delay after hypoosmotic exposure at 10 and 15 min, and start to decline at 20 min. **(C)** The fitted K^+^ currents are shown by the same types of line as in **(A)**. They appear with a greater delay at 15 min, 20 min, and 30 min. **(D)** The fitted background currents at same times as in **(A)**. Note the different scales in **(A)**, **(B)**, **(C)**, and **(D)**. While the K^+^ currents are smaller than the Cl^-^ currents, they persist for a longer time, up to 60 min after hypoosmotic shock (the figure was adapted in color from Beilby and Casanova, 2013).

As turgor regulation in *Lamprothamnium* was mostly achieved by varying K^+^ and Cl^-^ in the vacuole (Bisson and Kirst, 1980a), the increase of conductance must be due to the eﬄux of these ions. Okazaki and Iwasaki (1992) found a good correlation between Cl^-^ eﬄux and rise in conductance. Beilby and Shepherd (1996) employed the I/V technique and pharmacological dissection (TEA or LaCl_3_) to resolve the timing of the Cl^-^ and K^+^ outflow (see **Figure 5**). The large G K^+^ channels, identified by their typical I/V characteristics (compare top part of **Figures 2** and 5B) and total block by TEA, mediate the K^+^ outflow (Beilby and Shepherd, 2001b). The K^+^ channel activation is clearly visible with the Cl^-^ currents blocked by La^3+^ (**Figure 5B**). The opening of K^+^ channels is preceded by the Cl^-^ channel activation, but there is some overlap (Beilby and Shepherd, 1996). These results also suggest that K^+^ channels do not require increased Ca^2+^ concentration in the cytoplasm. By Occam’s razor argument the Cl^-^ channels are assumed to be the same Ca^2+^-activated channels that participate in the AP (Beilby, 2007, see also **Figure 3**). With the Ca^2+^ concentration in the cytoplasm high for many minutes (Okazaki et al., 2002), the I/V characteristics of the Cl^-^ channels could be investigated (see **Figure 5C** and Beilby and Shepherd, 2006b). The channels are inwardly rectifying with maximum conductance near -100 mV and strong inactivation near 0 PD. Bisson et al. (1995) found that in *C. longifolia* K^+^ channel activation preceded the Cl^-^ channel activation and that this second stage also required external Ca^2+^.

In both Characeae, there was an initial depolarization upon the hypoosmotic step, which was independent of Ca^2+^ concentration in the medium, or presence of the blockers TEA and La^3+^ (Bisson et al., 1995; Beilby and Shepherd, 1996). This response could be modeled by changing the reversal PD for the background current (see **Figure 5D**). Thus at least some of this current might flow through stretch-activated (mechano-sensitive) channels (Beilby and Shepherd, 1996; Shepherd et al., 2002).

### Hypoosmotic Effect Modulation by Cell Structure and Age

**FIGURE 6:**
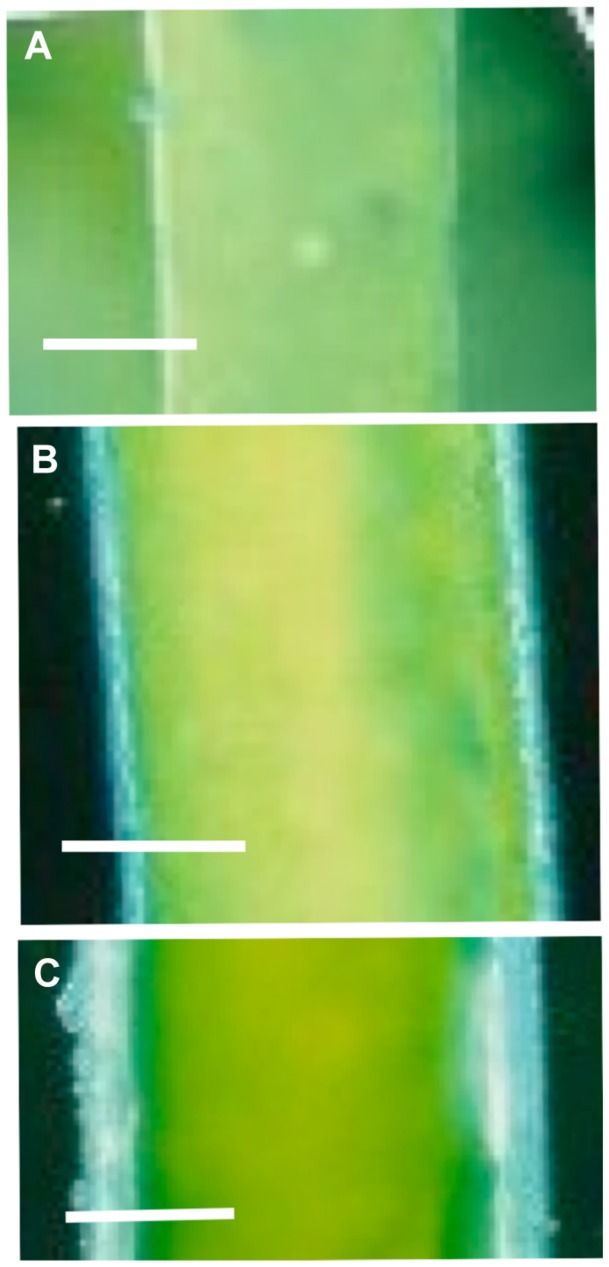
**The extracellular mucilage produced by *Lamprothamnium* plants**. Cells were stained with Alcian Blue at pH 1 (Beilby et al., 1999). **(A)** Apical cell growing in ¡ ASW with mucilage ∼7 μm thick. The staining is patchy, indicating that only fraction of the mucilage is sulphated. Bar = 50 μm. **(B)** Third internode from the apex from a plant growing in full ASW. Mucilage is ∼28 μm. Bar = 100 μm. **(C)** Seventh internode of the same plant as in **(B)**, mucilage thickness is ∼43 μm. Bar = 100 μm (from Beilby and Casanova, 2013).

Bisson et al. (1995) found that small cells (less than 10 mm in length) of *C. longifolia* regulated turgor within 60 min, while longer (and mostly older) cells took up to 3 days for full regulation. Beilby et al. (1999) and Shepherd and Beilby (1999) discovered that older *Lamprothamnium* cells developed coating of sulphated polysaccharide mucilage, identified by staining with Toluidine Blue or Alcian Blue at pH 1.0 (see **Figure 6**). With thicker mucilage the cells exhibited graded response to hypoosmotic challenge. The inflow of Ca^2+^ and streaming stoppage was not observed, but the opening of K^+^ channels was retained (such as **Figure 5B**). Very mucilaginous cells exhibited only a brief depolarization with linear I/V profiles, modeled as the background current with depolarizing reversal PD (**Figure 5D**; Shepherd et al., 1999). These cells still regulated turgor, but took 24 h or longer. Interestingly, Stento et al. (2000) were not able to find mucilage on cells of *C. longifolia* and it is possible that *Chara* genera do not produce it (Casanova, personal communication). On the other hand, Torn et al. (2014) collected six *Lamprothamnium* species from nine Australian locations and all species produced extracellular mucilage, with thickness and proportion of sulphated polysaccharides increasing with cell age, unrelated to the salinity of the environment.

After treatment of the mucilaginous cells with the heparinase enzyme, the cells responded to hypotonic shock with exaggerated depolarization, streaming stoppage and conductance increase (Shepherd and Beilby, 1999). Interestingly, the mucilage layer remained in place after heparinase treatment, but did not stain at low pH. Thus the effects of mucilage as an unstirred layer and a polyanionic layer could be separated. The removal of heparinase restored the muted response in the same cell. Young cells with no mucilage showed no change upon exposure to heparinase.

The young (fast regulating) and the old (mucilaginous and slow regulating) cells exhibited differences in sequestration of fluorochrome 6-carboxyfluorescein (6CF), which accumulated in the cytoplasm of the fast regulating cells and in the vacuole of the slow regulating cells. Beilby et al. (1999) speculated that the fast cells have more complex vacuole structure with canalicular elements, while slow cells had large central vacuoles. Patch clamp experiments performed on cytoplasmic droplets (thought to be bound by the tonoplast membrane) from slow and fast regulating cells exhibited K^+^ channels and small conductance Cl^-^ channels. The Cl^-^ channels appeared more active in slow regulating cells.

The ability of *Lamprothamnium* sp. to make extracellular sulphated polysaccharide mucilage (similar to that found in many chlorophyte marine algae) highlights another important halophytic attribute. Aquino et al. (2011) found that glycophytes of agricultural importance (*Zea mays* L., *Oryza sativa* L., and *Phaseolus vulgaris* L.) were unable to synthesize sulphated mucilage in their roots and leaves, even when challenged by increased salinity. On the other hand, salt tolerant angiosperms, such as mangroves and sea grass, and salt tolerant fern *Acrostichum aureum* contained sulphated mucilage in roots and shoots. Further, the seagrass *Ruppia maritima* L. only produced sulphated mucilage in saline media, even if the plants were supplied with abundant sulfate in the freshwater growth medium.

## Pathology of Salt Stress: Salt Sensitive Characeae

**FIGURE 7:**
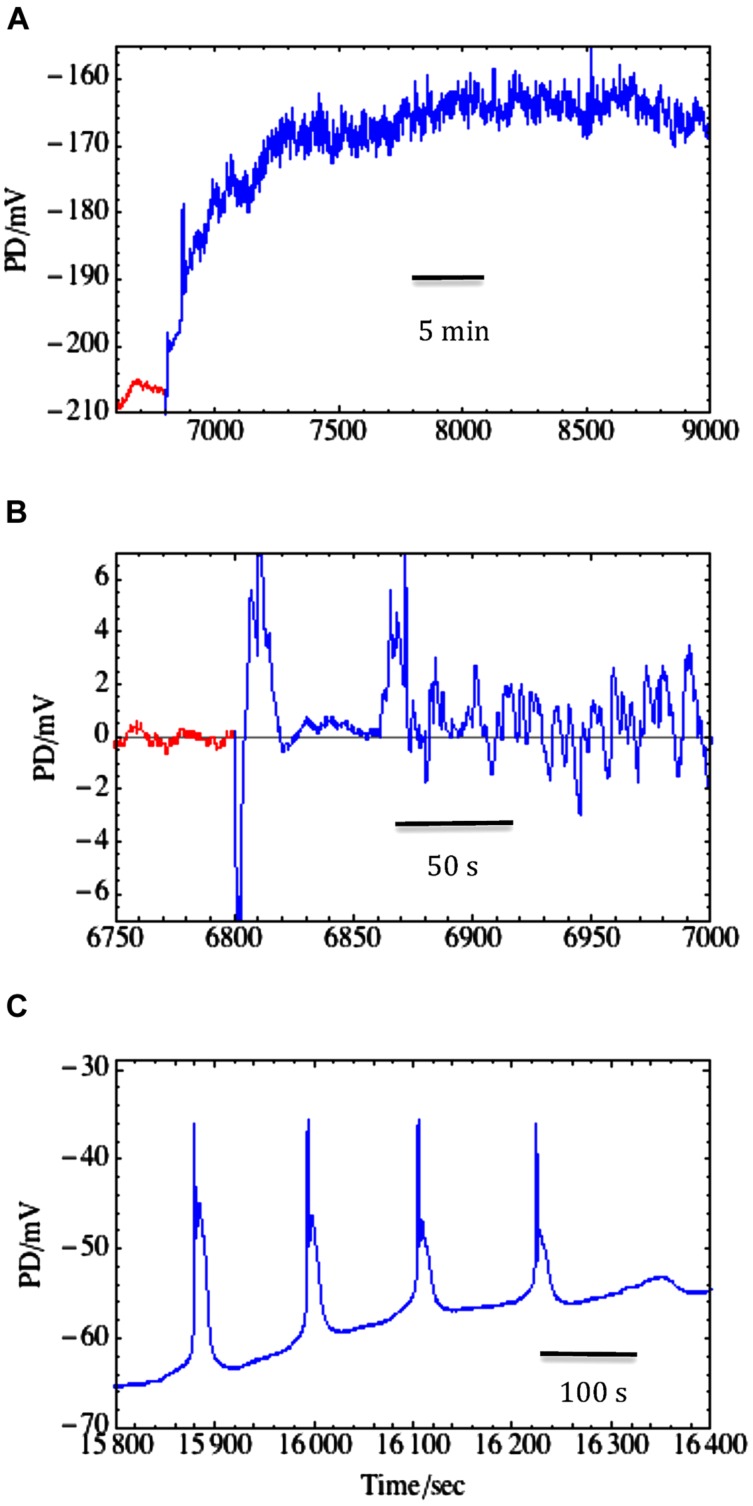
**Salinity-induced noise in membrane PD. (A)** Transition from Sorbitol APW (red) to 50 mM NaCl saline APW (blue) resulted in depolarization and noisy membrane PD. There was no AP, but the resting PD measurement was disrupted by medium change and exhibited spikes between 6800 and 6900 s. **(B)** Using the same trace on expanded time scale, the higher frequency noise was isolated by subtracting the running average (fitted with *n* = 100): noise in sorbitol APW (red) and saline-induced noise (blue) that started promptly after ∼50 s of the medium change (data from Beilby et al., 2014). **(C)** Spontaneous and repetitive APs after ∼80 min of Saline APW (Shepherd et al., 2008).

The salt sensitive *C. australis* was also exposed to the components of saline stress: sorbitol medium and saline medium of equivalent osmolarity. Shepherd et al. (2008) used 50–100 mM NaCl added to artificial pond water (APW) or 90–180 sorbitol APW. The proton pump in *Chara* cells does not respond to non-plasmolysing decrease in turgor, but can be transiently activated by an increase in Na^+^ concentration if Na^+^/Ca^2+^ ratio is not too high (Beilby and Shepherd, 2006a) and rapidly inactivated when Na^+^/Ca^2+^ ratio increases (Shepherd et al., 2008). The increase in Ca^2+^ concentration in saline media exerts its protective influence by partially blocking NSCC channels and keeping the pump running (Bisson, 1984). The two calcium effects may be related through cytoplasmic Na^+^ concentration, which may increase past some level critical to the pump. However, even at low external Na^+^ (2–3 mM) the lack of Ca^2+^ caused decline of the pump activity within hours (Bisson, 1984). The inactivation of the pump brings the membrane potential to E_background_, near -100 mV and at the excitation threshold. Spontaneous repetitive APs with long duration are often observed, further depleting the cell of K^+^ and Cl^-^ (see **Figure 7C** and Shepherd et al., 2008).

The background conductance does not change upon non-plasmolysing turgor decrease (as in *Lamprothamnium*), but increases in a Ca^2+^ dependent manner in saline media (Shepherd et al., 2008). Given equivalent salt stress the background conductance is higher in *C. australis* than in *Lamprothamnium* (Beilby and Shepherd, 2006a), as low turgor was found to increase the Na^+^ influx (see earlier section on Na^+^ transport).

Upon transfer from sorbitol to saline medium *Chara* exhibits salinity-induced noise in the membrane PD (see **Figures 7A,B** and Al Khazaaly et al., 2009). At frequencies between 1 and 500 mHz classical noise analysis revealed (1/f^2^) rise of noise power as frequency falls, and a marked increase in noise power upon salinity challenge. Inspection of the time domain shows a continuous but random series of small rapid depolarizations followed by recovery (**Figure 7B**). As PD noise is unchanged if NaCl is exchanged for Na_2_SO_4_, we initially hypothesized that high Na^+^ concentration activates H^+^/OH^-^ channels. However, in recent experiments (Eremin et al., 2013) employed fluorescent probe dihydrodichlorofluorescein (DCHF) to trace reactive oxygen species (ROS) formation under strong spot illumination of *Chara* surface. The authors suggest that excess ROS formed in the chloroplasts was carried away by the cytoplasm, oxidizing either histidine or sulfhydryl (SH) groups on transport proteins, leading to opening the H^+^/OH^-^ channels. In the animal kingdom, the voltage-gated H^+^ channels in the brain microglia are activated by H_2_O_2_ (Wu, 2014). Beilby et al. (2014) were able to inhibit or postpone the saline-induced noise by presoaking the *Chara* cells in strong antioxidant melatonin prior to saline stress. Therefore, H^+^/OH^-^ channels may be activated by an oxidative burst upon exposure to saline. Such a transient increase in ROS was observed by Li et al. (2007) upon exposing rice roots to salinity.

**FIGURE 8:**
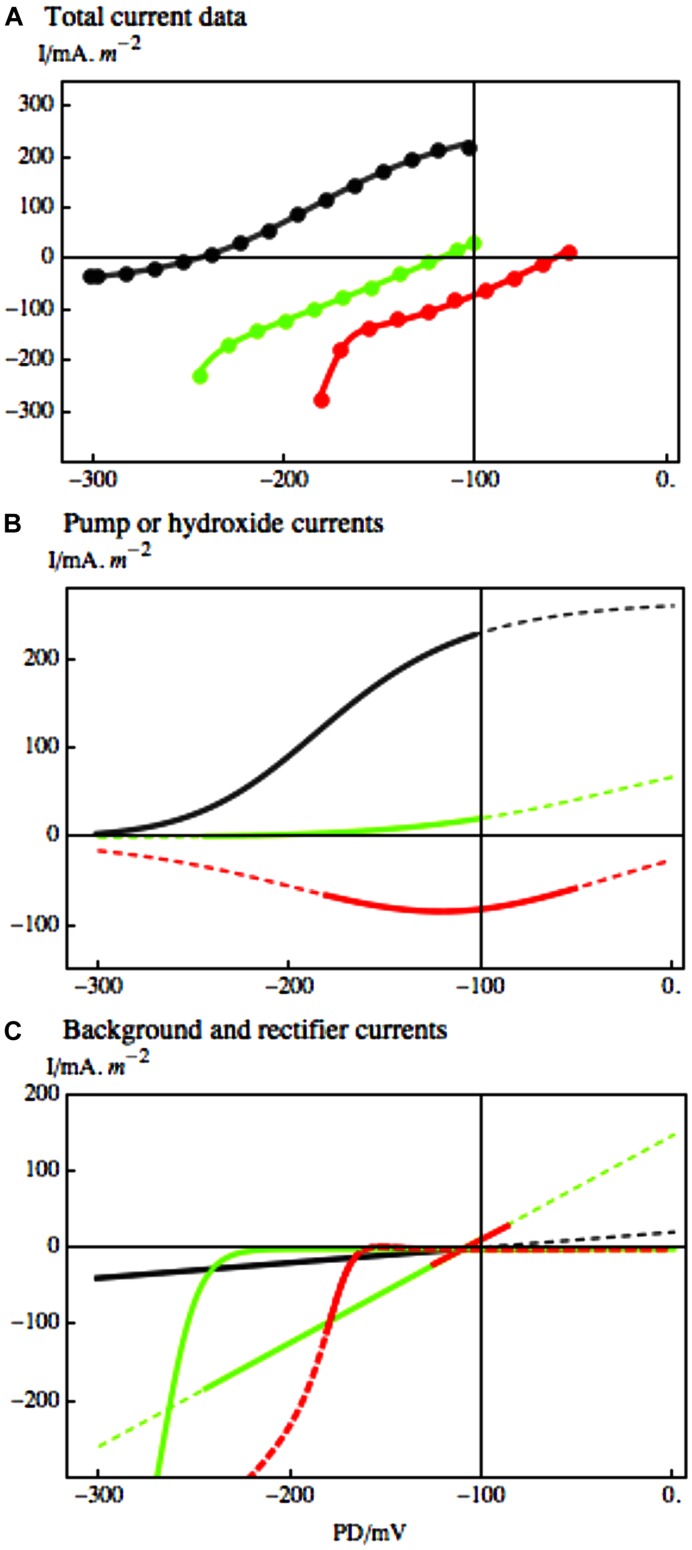
**The response of *Chara* I/V characteristics to salinity challenge. (A)** Pump-dominated profile (black) in Sorbitol APW evolved to background-dominated profile after 67 min in 50 mM NaCl Saline artificial pond water (APW; green) and to upwardly concave profile (red) after 117 min of Saline APW. The experimental data, shown as points in **(A)**, were fitted by the pump or OH^-^ channel models **(B)** and background current and inward rectifier models **(C)**, using same line colors as in part **(A)**. The parameters are given in caption of Figure 1 of Beilby and Al Khazaaly (2009). The dashed lines in **(B)** and **(C)** extrapolate the models beyond the range of the data. The inward rectifier channels also activate at more depolarized PDs, but the membrane PDs are more depolarized for K^+^ inflow to take place.

Once again the modeling of the I/V data allowed us to trace responses of ion transporter populations as function of exposure to saline. With longer exposure to high salinity, the membrane PD of *Chara* cells continues to depolarize toward zero, while the noise diminishes (suggesting that progressively larger numbers of H^+^/OH^-^ channels were activated – Beilby et al., 2014). The shape of the I/V characteristics changes and could be simulated by H^+^/OH^-^ channels and increased background current (see **Figure 8** and Beilby and Al Khazaaly, 2009). The global opening of these channels at the time of saline stress would be disastrous for the cells, as both the negative membrane potential and the pH gradients between the cytoplasm, vacuole and the medium are necessary for the cell survival (**Figure 3**).

**FIGURE 9:**
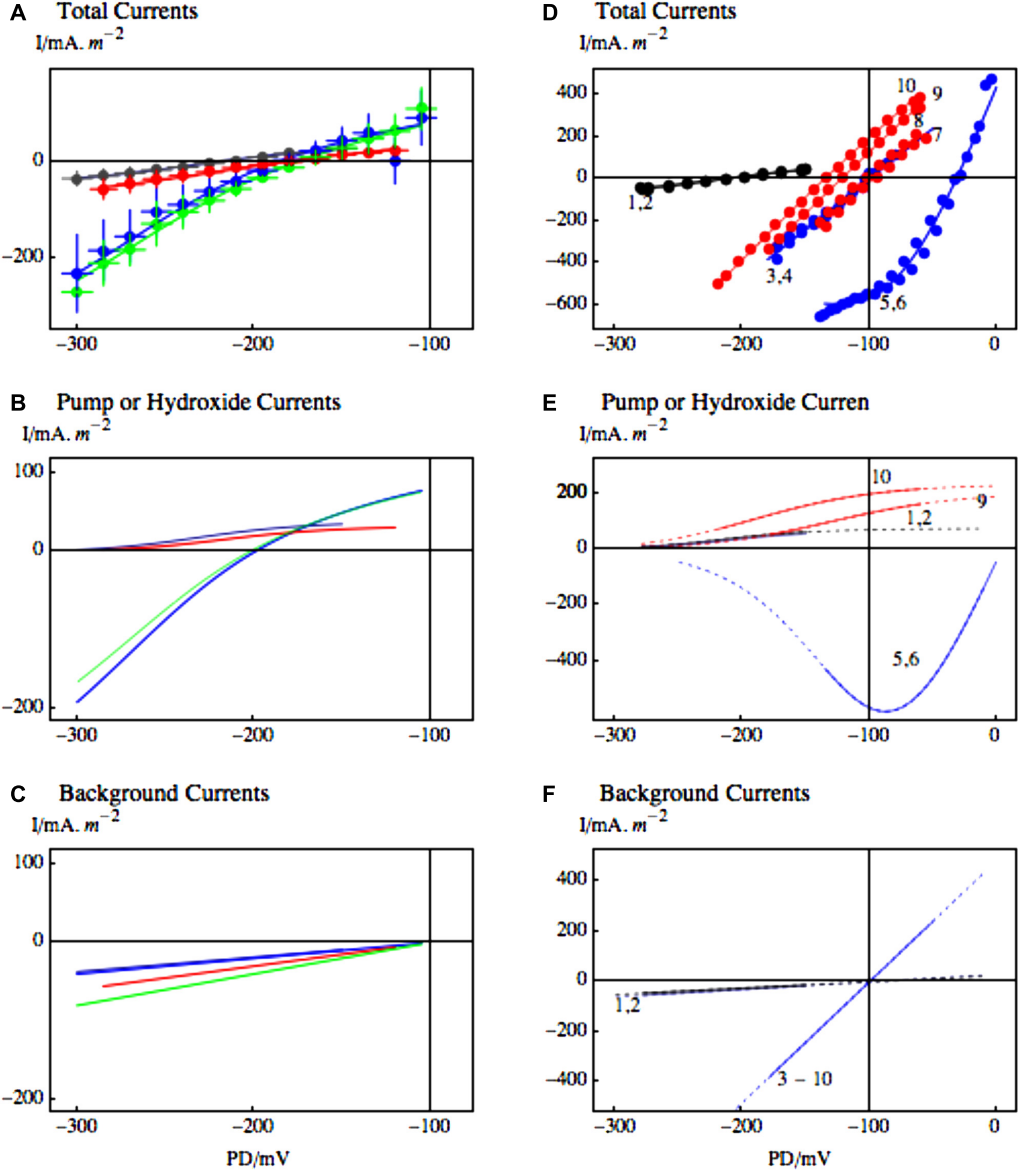
**The effect of zinc ion on the high pH state and putative H^+^/OH^-^ channels at the time of salinity stress. (A)** Statistics of 12 I/V profiles from 5 cells in APW (black), APW with pH increased to 11 (blue), 1.0 mM ZnCl_2_ was then added to the high pH APW for average time of 36 min (red) and finally in three cells where 0.5 mM 2-mercaptoethanol (ME) replaced ZnCl_2_ for average 35 min after the high pH state was inhibited (green). The data were fitted with the pump current or the OH^-^ current **(B)**. The background current increased slightly after application of ZnCl_2_ **(C)**. The fit parameters are given in Al Khazaaly and Beilby (2012). **(D–F)** The effect of zinc ion on salinity induced I/V profiles. **(D)** The profiles 1 and 2 (black) were obtained in APW and APW with 90 mM sorbitol. After 15–30 min of APW + 50 mM NaCl, the I/V profiles 3,4 just showed the background current (blue). The resting PD then dropped further and the I/V profiles 5 and 6 were modeled with OH^-^ channels (blue). 1.0 mM ZnCl_2_ was added to the saline APW and the PD repolarized to the background current (I/Vs 7 and 8, red) and later the pump was re-activated (I/Vs 9 and 10, red). For details see **Figures 2** and 4 of Al Khazaaly and Beilby (2012).

Further evidence for role of H^+^/OH^-^ channels in the salt stress pathology is their blockage by zinc ion (Al Khazaaly and Beilby, 2012). Zinc ions are the most potent inhibitors of animal proton channels. While the permeating ion in Characeae is more likely OH^-^, the channel proteins may still be closely related, as replacement of aspartate 112 by a neutral amino acid facilitated anion conduction in animal “proton” channels (DeCoursey, 2013). In Characeae the zinc ion reversibly inhibited the high pH state. The application of 0.5 mM 2-mercaptoethanol (ME) removed the zinc and restored the high pH state (Figures 9A–C). At the time of salt stress the depolarization to PD levels above -100 could be reversed by including 1.0 mM ZnCl_2_ in the saline APW. Even the function of the proton pump was temporarily restored (Figures 9D–F). The saline noise was also inhibited by zinc ion. However, as zinc has many functions in plant tissues, further proof of H^+^/OH^-^ channel involvement in salt stress pathology is needed.

Interestingly, Kirst and Bisson (1982) found that salt tolerant *Lamprothamnium* exposed to pH above 9.5 suffered similar fate to salt sensitive *C. australis*: the turgor dropped, concentration of Na^+^ in the cell compartments increased, while concentration of K^+^ and Cl^-^ decreased leading to cell death. We assume that high pH opened the H^+^/OH^-^ channels, placing the *Lamprothamnium* cell in the same electrophysiological state as *Chara* in the late stages of salt stress.

## Conclusions, Relevance to Higher Plants and Future Research

The salt tolerant and salt sensitive Characeae contain the same types of ion transporters (**Figure 3**), but some of these transporters respond differently to osmotic and saline stress. The salt tolerant *Lamprothamnium* and *C. longifolia* cells sense both the decrease of turgor and the increase of Na^+^, and respond by pumping protons faster to maintain a negative membrane potential while keeping H^+^/OH^-^ channels closed in the acid bands. (The pH banding phenomenon as a function of salinity is under investigation at present. pH banding was observed in *Lamprothamnium* cells acclimated to fresh water – Beilby, unpublished). The cells regulate turgor by importing more K^+^, Cl^-^ and Na^+^. Salt sensitive *C. australis* does not respond to turgor decrease, does not regulate turgor, loses the energizing pump function and negative membrane potential and undergoes spontaneous repetitive APs. The global opening of H^+^/OH^-^ channels speeds up the irreversible decline by further decreasing the membrane PD and promoting K^+^ loss through outward rectifying channels. The proton gradient powering Na^+^/H^+^ antiporter and 2H^+^/Cl^-^ symporter is dissipated.

Is the H^+^/OH^-^ channels and the proton pump combination just peculiar to the ancient Characeae? This complex electrophysiological motif of pH banding can be observed in higher plants: aquatic angiosperms (Prins et al., 1980), pollen tubes (Feijo et al., 1999) and, most importantly, roots of land plants (Raven, 1991). In roots the source of the current is located in the acid root subapical zone with the sink at the alkaline tip (Raven, 1991). The author suggests that the acid and alkaline zones facilitate acquisition of molybdenum, phosphorus and iron as well as reduction of aluminum toxicity. Protoplasts from wheat roots were found to change from pump-dominated to H^+^/OH^-^ channel dominated state (Tyerman et al., 2001). Further, salinity induced noise was observed in wheat root protoplasts (Tyerman et al., 1997). Thus a future experiments should investigate the effect of salinity on the acid/alkaline zones of roots of both glycophytes and halophytes.

Being able to sense turgor is clearly important for salt tolerant cells. Staves et al. (1992), Shimmen (2008) suggest that it is the nodal complexes (see **Figure 1**) of the characean cells that sense difference in turgor. Future experiments are planned with node-less constructs (Beilby and Shepherd, 1989, 1991) from *Lamprothamnium* cells to find if these can still sense turgor and regulate it.

How do the turgor sensors and Na^+^ sensors communicate with the proton pump? Are the proton pumps of salt tolerant and salt sensitive Characeae different? Plant H^+^ ATPase is encoded by a multi-gene family. In rice a new isoform of the proton pump genetic family was observed in response to salt stress (see a review by Janicka-Russak, 2011). This isoform was similar to that found in halophyte *Suaeda maritima*. Several post-translational modifications are also suggested, involving C-terminal domain and N-terminus of the protein. Phosphorylation is regulated by 14-3-3 proteins and pump molecules might form multimeric complexes. In electrophysiological experiments the pump current can be observed directly as function of time after osmotic or salinity increase. It might be possible to probe the post-translational modifications in response to salinity stress. The imminent sequencing of *C. braunii* genome (Stefan Rensing, personal communication) will provide molecular data of proton pump structure in salt tolerant and salt sensitive Characeae.

## Conflict of Interest Statement

The author declares that the research was conducted in the absence of any commercial or financial relationships that could be construed as a potential conflict of interest.
